# Development and Application of Novel Caregiver Hygiene Behavior Measures Relating to Food Preparation, Handwashing, and Play Environments in Rural Kenya

**DOI:** 10.3390/ijerph15091994

**Published:** 2018-09-13

**Authors:** Breanna K. Wodnik, Matthew C. Freeman, Anna S. Ellis, Emily Awino Ogutu, Amy Webb Girard, Bethany A. Caruso

**Affiliations:** 1 Hubert Department of Global Health, Rollins School of Public Health, Emory University, Atlanta, GA 30322, USA; awebb3@emory.edu; 2 Department of Environmental Health, Rollins School of Public Health, Emory University, Atlanta, GA 30322, USA; matthew.freeman@emory.edu (M.C.F.); anna.s.ellis@emory.edu (A.S.E.); emily.awino@yahoo.com (E.A.O.); bethany.caruso@emory.edu (B.A.C.)

**Keywords:** stunting, handwashing, food hygiene, play environment, animal feces, factor analysis

## Abstract

Exposure to fecal pathogens results in both acute and chronic sequalae in young children. Diarrhea causes nearly 20% of all under-five mortality, while even sub-clinical enteric infections may lead to growth shortfalls. Stunting affects nearly 165 million children globally and results in lifelong and intergenerational effects for the world’s poorest populations. Caregiver hygiene behaviors, such as those surrounding handwashing and food preparation, play a critical role in exposure to fecal pathogens; standard metrics to assess these behaviors are warranted to provide a means of quantifying the impact these behaviors have on enteric infections and to evaluate the success or failure of interventions and programs. This paper documents the development of three novel caregiver hygiene behavior measures: hygienic food preparation and storage, handwashing at key times, and provision of a safe play environment for children under two years. We developed these measures using formative qualitative work, survey creation and deployment theoretically underpinned by the COM-B model of behavior change, and exploratory and confirmatory factor analysis. The final measure for hygienic food preparation and storage includes 10 items across two factors; the final measure for handwashing at key times includes 15 items across three factors; and the final measure for safe play environment contains 13 items across three factors. Future researchers may employ these measures to assess caregiver behaviors in other populations, identify specific behavioral dimensions that should be the focus of interventions, and evaluate interventions and programs.

## 1. Introduction

Exposure to fecal pathogens results in both acute and chronic sequalae in young children. Diarrhea causes 1.4 million deaths annually, nearly 20% of all under-five mortality [1,2]. Along with diarrhea, even persistent sub-clinical enteric infections may lead to environmental enteric disfunction (EED) and growth shortfalls [3]. Stunting, defined as a length- or height-for-age more than two standard deviations below normal, affects an estimated 165 million children globally [4,5].

Major determinants in a child’s growth include access to healthcare, food access, incidence of diarrheal diseases, maternal nutrition, feeding practices, and access to safe water and basic sanitation [6,7]. Recent studies have found limited efficacy of water, sanitation, and hygiene (WASH) interventions on the outcome of child stunting [8,9,10], although hygiene behavior change is influential over a wide variety of health outcomes. Good hygiene practices may be influential in affecting health outcomes closely related to growth stunting, through reducing the incidence of diarrhea and related morbidities and mortalities, the transmission of foodborne and zoonotic diseases, and EED [11,12,13]. 

The first 1000 days, from conception through the first 2 years of life, are critical windows both for child growth and to avoid enteric infections [14]. A mother’s behavior during her pregnancy and the caregiver’s behavior in early childhood can either mitigate or exacerbate the impact of modifiable environmental conditions on growth outcomes. Caregivers dictate or influence many exposures to a child in the first 1000 days of life [14], and while research has explored the biological and environmental factors that contribute to stunting [5,7,15,16,17,18,19], there is limited evidence on how caregiver behaviors may impact the outcome of stunting in their children in low income settings. 

One challenge in studying environmental conditions or caregiver behavior is the measurement of those conditions and behaviors. As the empirical outcomes for many of these behaviors are themselves difficult to accurately quantify, measurement of latent, unobservable variables such as social influences and perceived capabilities can be a useful tool to detect change over time. As Menon and Frongillo note in response to the findings from the WASH Benefits trials in Bangladesh and Kenya [20], caregivers create enabling microenvironments for children comprised of thousands of behaviors, from feeding to cleaning, and there is a need to focus on the parent and child dyad to identify how best to support caregiving behaviors that promote child growth and development. To that point, the aim of this paper is to describe the development of novel measures to assess drivers of three key caregiver behaviors related to hygiene: hygienic food preparation and storage, handwashing at key times, and provision of safe play environments for children. Informed by the development of food, water, and sanitation insecurity measures [21,22,23,24,25], we used a theory-driven approach and the COM-B model [26] to explore the capabilities, opportunities, and motivations underlying the behaviors related to caregiver hygiene practices in rural Kenya. The COM-B framework is so named because it summarizes the following three pre-requisites for behavior change: capability (the person has the skills necessary to perform the behavior), opportunity (there are not existing social or environmental constraints that hinder performance of the behavior), and motivation (the person has strong personal and external reasons to perform the behavior). Understanding and assessing these behavioral antecedents through use of these novel quantitative measures can provide a baseline understanding of caregiver behaviors and identify potential points for improvement. These three hygiene measures may be used alone or in conjunction to assess the impact of interventions that are targeting outcomes in food preparation and storage, handwashing at key times, and provision of safe play environments in children.

## 2. Materials and Methods 

### 2.1. Setting

This project is located in the Homa Bay and Migori Counties of western Kenya. Measures for behavioral antecedents to outcomes of stunting are particularly relevant in this region, as 35.3% of children are stunted in Kenya [27]. Data were collected in June and July of 2017 via a household survey of mothers participating in neighbor women’s groups, the purpose of which was to assess household conditions and caregiver capability, opportunity, and motivation related to selected WASH and nutrition outcomes of interest. 

This study was conducted within the broader context of an assessment of a demand-side, integrated WASH, early childhood development, and nutrition behavior change intervention within the THRIVE II project. THRIVE II is led by Catholic Relief Services (CRS) and supports HIV/AIDS-affected children under the age of two years and their caregivers in parts of Kenya, Tanzania, and Malawi. The purpose of the project is to create a sustainable culture of care, and to reinforce and inspire good caregiver behaviors around early stimulation, infant and young child feeding (IYCF), and WASH-related behaviors (publications forthcoming). Within that context, we identified a need for the ability to measure changes in behavior, thus, we developed a set of quantitative measures. We utilized the COM-B framework, a behavior change model developed by Michie, Atkins, and West in 2011, in the structuring of an intervention and evaluation strategy because of its coherent theory-to-intervention pathway using the innovative behavior change wheel, and because of its comprehensive inclusion of specific theory and evidence-based behavior change techniques and theoretical domains [26].

### 2.2. Overview of Study Design

**Figure 1:**
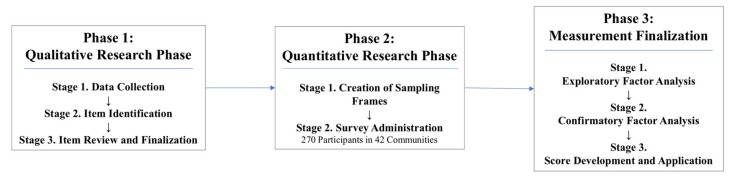
Flowchart for measurement development.

Our study design followed a sequential three-phase process that aligned with previous research in creating experienced-based measures [21,23,24,25], including a qualitative research phase, quantitative research phase, and a measurement finalization phase (Figure 1).

The research conducted in the qualitative phase was used to generate items for the measures. During the quantitative research phase we created a sampling frame and collected responses for the items via a household survey. Finally, during the measurement finalization phase, we explored the factor structure of the items by each individual measure via exploratory factor analysis (EFA), and confirmed the resultant structures via confirmatory factor analysis (CFA). The measures reported here are our finalized set of recommended items for hygienic food preparation and storage, handwashing at key times, and provision of safe play environment measures, and are accompanied by mean scores for this population. In the next section, we detail each research phase.

### 2.3. Phase 1: Qualitative Research 

The qualitative research phase was conducted in three stages: data collection, item identification, and item review and finalization.

#### 2.3.1. Phase 1, Stage 1: Data Collection

Qualitative research was conducted from September to December of 2016. First, a team of CRS and Emory researchers conducted direct structured observation of caregiver hygiene behaviors and feeding practices in 12 households. We then conducted a total of 24 focus group discussions with mothers, fathers, and grandmothers, all of whom serve significant roles in caring for children under two years of age (CU2) in Kenya. Twenty-nine key informant interviews were also conducted with religious and community leaders, community health volunteers (CHVs), and community health extension workers (CHEWs). Details on this formative research methodology and the results are forthcoming.

#### 2.3.2. Phase 1, Stage 2: Item Identification

The formative research primed the development of a set of problem and solution trees [28], which were constructed with stakeholders and CRS staff during an intervention design workshop in Kenya. The solution trees outlined intervention points for caregiver behaviors that were aligned with the THRIVE II behavior change strategy and which local stakeholders believed to be the most amenable to change. These key behaviors were directly transferred to a logical framework (logframe), a tool we used to develop the baseline survey in a systematic way that organized the evaluation from activity level to the impacts the program aimed to achieve [29]. 

The COM-B domains and theoretical domain framework (TDF) served to link the formative work to the intervention functions [26] and corresponding logframe. From the logframe, we developed a total of 61 items which reflect the capability, opportunity, and motivational barriers and facilitators for behaviors that may influence the outcome of stunting in children. Those items were categorized as being associated with specific outcomes related to caregiver behaviors: responsive feeding practices, preparation of porridge of sufficient caloric density [30], hygienic food preparation and storage, knowledge of handwashing at key times, or provision of a safe play environment to CU2. Since, to our knowledge, no definitions previously existed for the primary hygiene outcomes of interest, our team developed a set of operational definitions based on context from the qualitative formative work (Table 1).

#### 2.3.3. Phase 1, Stage 3: Item Review and Finalization

Items were reviewed by all co-authors, CRS directors and staff, and a team of four research assistants (RAs) prior to piloting in order to assess content validity. Reviewers’ comments were mainly used to edit the wording of existing items, although two items related to discarding food that had been left sitting out or uncovered were rejected entirely based on a shared perception that all respondents would unanimously strongly disagree. The four RAs then translated the items to Luo in teams of two, then read the translations aloud to the other team to assess face validity.

The finalized 61 items followed a five-point ‘agree/disagree’ Likert scale (1 = strongly disagree, 2 = somewhat disagree, 3 = neither agree nor disagree, 4 = somewhat agree, 5 = strongly agree). Piloting of the items in a community similar to those in which data collection would take place was used as a final check for face validity. The RAs found that the items themselves were well-understood and accepted, but noted some difficulties in explaining and translating the Likert scale itself, a commonly-faced challenge in quantitative research [31]. Author E.A.O. and the RA team, all fluent in both English and Luo, worked together to create a stronger explanation of the scale and adjusted translation slightly for the terms ‘somewhat disagree’ and ‘somewhat agree’. The re-translated Likert scale was reviewed and back-translated by Luo-speaking CRS staff to ensure correct translation.

### 2.4. Phase 2: Quantitative Research

Quantitative research was conducted in two stages: creation of sampling frames, and survey administration.

#### 2.4.1. Phase 2, Stage 1: Creation of Sampling Frames

The survey was conducted from June to July of 2017 in 42 communities with members of neighbor women’s groups, as part of the care group model [32] employed by THRIVE II. Neighbor women’s groups (NWGs) were randomly selected from a full list of THRIVE II participating villages, NWGs, and their participants provided by partnering organizations Homa Hills Community Development Organization (HHCDO) in Homa Bay and Mercy Orphans in Migori. No two selected NWGs were in the same community, and all had a minimum of eight participating neighbor women. Survey participants were required to meet the following criteria to be eligible for the baseline survey: (1) must be a member of a THRIVE II neighbor women’s group; (2) must be 18 years or older; and (3) may not be a CGV. While CGVs are part of the NWGs, they were excluded from the baseline survey because they receive additional trainings and education above that of the other non-CGV neighbor women. The total number of women selected for participation was 352 (120 in Homa Bay; 232 in Migori), with a 77% response rate for a total of 270.

The sample size was calculated for the overall study assessing changes in key behavioral outcomes. Accounting for the clustered design, the total calculated sample size estimate was 276 participants [33]. For the purposes of measurement development and analysis, 10 participants per item is commonly used to estimate sample size [34]; these estimations were therefore also taken into consideration based on the handwashing at key times outcome, which has the largest number of items (15), resulting in an estimation of roughly 300 participants (150 per factor analysis arm).

#### 2.4.2. Phase 2, Stage 2: Survey Administration 

The survey instrument was piloted in non-study communities to determine readability and cultural appropriateness. Trained enumerators collected data on the 61 items, as well as participant demographics (Table 2), WASH behavior and access, diet diversity for mother and child using the FAO 24-hour recall strategy [35,36], and animal presence and caretaking behavior in a one-hour survey (Table 3). The household food insecurity access scale (HFIAS) composed of nine items and three domains was used to measure household food insecurity in the previous 30 days [22]; for this scale we asked only HFIAS occurrence questions without including frequency follow-up questions. Survey participants were given a verbal explanation of the five-point ‘agree/disagree’ Likert scale for our items, accompanied by a physical card with the scale written out in Luo.

Physical observations were conducted of the household and surrounding compound at the time of the survey. Observed features included the physical space in which food is prepared and utensils used for food preparation; storage of previously-prepared food intended for future consumption (if present at time of observation); handwashing stations or locations within the compound; the primary space in which the CU2 plays or spends the day; and visible cleanliness of the child’s hands (presence of dirt on palms, fingerpads, under fingernails, and length of fingernails) (operational definitions in Table 1). As part of their training, enumerators individually observed the same household features and compared their survey responses to ensure consistency.

Enumerators were accompanied by a CGV to improve acceptance into the community and to provide direction to the participants’ households. We utilized the Open Data Kit (ODK) system for electronic data collection. Cell phones used for surveying were purchased for the sole purpose of survey data collection. All completed surveys were loaded daily to a secure database; data collected was stored in a password-protected file.

### 2.5. Phase 3: Measurement Finalization

The measurement finalization phase involved three stages: exploratory factor analysis, confirmatory factor analysis, and score development and application. MPLUS7 software (Muthén & Muthén, Los Angeles, CA, USA) was used for all factor analysis; all other survey data were analyzed using Stata Statistical Software: Release 15.1 (StataCorp LP, College Station, TX, USA).

#### 2.5.1. Phase 3, Stage 1: Exploratory Factor Analysis 

EFA is used to explore the relationships within a set of data and CFA used to further confirm those relationships, such that patterns within a set of variables may be both more easily understood and interpreted [38]. EFA is the first step if there has not been prior research conducted on the topic, with CFA used to test the resultant factor structure [39]. 

The total sample was randomly split to enable half the data to be used for EFA and the other to confirm the resulting factor structure with CFA (n_EFA_ = 135; n_CFA_ = 135). There was no more than 10% difference between subpopulations in the datasets based on county of residence, education level, and having a CU2. 

Descriptive statistics were generated for all items to check for potential outliers and non-normal data by observing the distribution, skewness, and kurtosis of responses to each item (Supplementary Tables S1–S4). While it is not required for EFA that all items be normal, it may have a substantial effect on the EFA results because variables which are too highly correlated may form artefactual factors [39].

EFA was conducted to explore individual factor structure for each of the five behaviors separately. It was carried out with 21 items for hygienic food preparation and storage, 15 items for handwashing at key times, 14 items for provision of safe play environment, 4 items for porridge thickness, and 7 items for responsive feeding practices. Factor structures could not be determined for responsive feeding and porridge thickness due to a small number of items and very low variability in responses; those behaviors were therefore dropped from further exploration and we focused our research on the remaining hygiene behavior factors. 

We hypothesized that, within each behavior, items would generally fall together by the COM-B structure. We chose the oblique PROMAX rotation as we believe the dimensions underlying these constructs and the variables that represent them are correlated; the weighted least square mean and variance (WLSMV) estimator was used as all variables are categorical [39,40]. As per the Kaiser–Guttman rule, we considered all factors with an Eigen value greater than one [41]. For the three remaining behaviors, we decided a priori to drop items if they were (1) too highly correlated with another item; (2) had a large negative residual variance that resulted in a non-positive definite covariance matrix; (3) the factor loading was <0.3 [40,41]. Had there been high cross-loaders (factor loadings >0.50 on each factor), those items would also have been dropped [41].

#### 2.5.2. Phase 3, Stage 2: Confirmatory Factor Analysis

CFA was used to test the factor structure achieved in EFA with the second subset of the data (n_CFA_ = 135). As with EFA, we assessed each behavior separately, utilizing the PROMAX rotation and a WLSMV estimator. The resulting factor loadings are listed as standardized solutions using the STD output (Table 4). The root mean square error of approximation (RMSEA) was used to assess model fit (as a rule of thumb, RMSE A ≤ 0.06 indicates a good model fit; RMSEA ≤ 0.08 indicates moderate fit; RMSEA ≥ 0.1 indicates poor fit) in conjunction with the comparative fit index (CFI) and the Tucker–Lewis index (TLI), where a value ≥ 0.95 indicates a strong model fit for both CFI and TLI [42]. No further items were dropped from the factor structures at the CFA stage.

#### 2.5.3. Phase 3, Stage 3: Score Development and Application

Measurement scores were calculated using the factor structure determined by EFA and confirmed by CFA. The sum of the responses from the Likert scale were calculated by the resultant factor structure, and divided by the number of items within that factor. Finalized scores for individual respondents could range from 1–5, with a greater mean frequency of occurrence reflected by higher scores [21]. The generated scores from the three hygiene measures were modeled against direct observations from the survey using two-sample *t*-tests to assess whether higher scores related to higher outcome of the performed behavior.

### 2.6. Ethical Approval

This study was approved by the Emory University Institutional Review Board (Atlanta, GA, USA; IRB00090057) as well as the National Commission for Science, Technology and Innovation (NACOSTI) Ethical Review Board on the Kenyan national level and the Great Lakes University of Kenya (GLUK) Ethical Review Boards on the Kenyan local level. Each participant was read a full consent form in Luo; consent was given orally. 

## 3. Results

### 3.1. Participant Demographics

A total of 270 women were surveyed for this study. All participants were part of a neighbor women’s group, nearly two-thirds (63%) had a child under the age of two, half were currently lactating (49%), and 11% were pregnant at the time of survey administration. Most participating women had not completed formal education beyond primary school (83%). Fewer than half (44%) of the participating households self-reported having access to a latrine, and roughly half (51%) reported using surface water as their primary source of drinking water. Food insecurity within HFIAS domains was very high among participating households, with nearly all households reporting some level of anxiety and uncertainty about food supply as well as having insufficient food quality or quantity in the previous 30 days [22] (Table 2).

Few households (12%) had a hygienic food preparation space by operational definition, and fewer than half of households (42%) were storing food hygienically. A quarter of the women (26%) were able to list five of six key handwashing times, and water and soap presence at handwashing stations was low (12% and 7%, respectively); nearly half (41%) of observed CU2 hands were clean, and self-reported handwashing with soap was high. About a third of households (34%) demonstrated an absence of human and animal feces, garbage, and other harms in the area that caregivers reported as the primary play location of the CU2 (Table 3). 

### 3.2. Hygiene Behavior Measure Survey Item Frequencies

Twenty-one items were initially analyzed for the food preparation and storage measurement. Items to which participants most often responded ‘strongly agree’ were those related to personal beliefs surrounding hygienic practices: “It is beneficial to wash food before preparation” (89%) and “It is beneficial to store food in a covered container” (88%). The food preparation and storage item to which participants most often responded ‘strongly disagree’ was: “It is okay to cut vegetables with the same knife just after I cut raw chicken or fish” (83%).

Fifteen items were initially analyzed for the handwashing at key times measurement. Those items to which participants most often responded ‘strongly agree’ related to physical opportunity for handwashing and perceived self-efficacy: “It is important for me to have soap available for handwashing” (89%); “I always have water for handwashing” (84%). The handwashing items to which participants most often responded ‘strongly disagree’ were social beliefs about handwashing practices widely in the community: “Most people in my community use soap every time they wash their hands” (20%); “Most people in my community wash their hands after defecating” (19%); “Most people in my community have soap” (18%).

Fourteen items were initially analyzed for the provision of a safe play environment measure. The items to which participants most often responded ‘strongly agree’ were those related to beliefs surrounding animal feces: “Dog feces can make you sick” (96%); “I find it disgusting when animal feces (including chicken feces) are present within a compound” (90%); “Chicken feces can make you sick” (84%). The safe play environment items to which participants most often responded ‘strongly disagree’ were related to the social aspects of what participants believed other members of their community were practicing: “Most people in my community have a designated play area for their young children” (39%); “Most children in this community play in areas that are free from garbage or other wastes” (26%) (see Supplementary Tables S1–S4 for frequencies of all responses).

### 3.3. Exploratory Factor Analysis

#### 3.3.1. Measure 1 EFA: Hygienic Food Preparation and Storage

A total of 11 items were omitted a priori from the hygienic food preparation and storage measure; the resultant 10-item, two-factor structure has strong theoretical fit and moderate statistical fit (RMSEA = 0.74). Three items (E1, E11, E59) were over-correlated with other variables in the solution, and were removed one-by-one and in that order. Five items (E61, E8, E60, E58, E3) were subsequently removed one-by-one due to large negative residual variances. Three items (E9, E15, E16) were deleted one-by-one, in that order, due to a failure to load onto any factors (factor loadings < 0.3) (see Supplementary Tables S1–S4 for a list of all initial items).

The two finalized factors are ‘social opportunity’, which contains items related to what the participant sees as normalized behaviors surrounding food hygiene practices within their community (factor loadings: 0.642–0.904), and ‘personal beliefs’, which includes items on best practices, food safety, and confidence relating to personal knowledge (factor loadings: 0.595–0.814).

#### 3.3.2. Measure 2 EFA: Handwashing at Key Times

All 15 handwashing at key times items loaded onto one of three factors with strong model fit in EFA (RMSEA = 0.055). The first factor, labeled as ‘physical opportunity’, contained three items related to access of soap, water, and sufficient time for handwashing (factor loadings: 0.363–0.824). The second factor, labeled as ‘social opportunity’, contained seven items relating to the participant’s perception of the actions of others in their community related to handwashing, including the timing of handwashing, possession and use of soap, and child handwashing behaviors (factor loadings: 0.662–0.936). The four items for the third handwashing at key times factor all related to the motivations behind the participant’s choice to wash or not wash their hands, which were labeled as ‘beliefs and self-efficacy’ (factor loadings: 0.359–0.942).

#### 3.3.3. Measure 3 EFA: Provision of Safe Play Environment

One item for the provision of safe play environment measure was omitted (item 26) due to its large negative residual variance, resulting in a non-positive definite matrix. The remaining 13 items presented a resultant three-factor structure, which was selected because, despite weak model fit (RMSEA = 0.118), it demonstrated a strong theoretical fit. The first factor was labeled as ‘perceptions around animal feces’ and included six items which related either to belief of illness related to animal feces or disgust factors surrounding feces in the household or compound (factor loadings: 0.424–0.682). The second factor contained four items related to beliefs surrounding the safe play environment practices for children in other households within the community and was labeled ‘social opportunity’ (factor loadings: 0.672–0.996). The final factor, labeled ‘reflective motivation’ and dealing with the perceived capability to provide a safe play environment for children, had three items (factor loadings: −0.864 to −1.001).

### 3.4. Confirmatory Factor Analysis 

No further items were omitted during CFA. The factor loadings for the final 38 items in each of the three measures were sufficiently large (>0.3). The statistical model fit for the handwashing at key times measure had a weak RMSEA (0.114; CI 0.097–0.132), but moderate CFI (0.934) and TLI (0.921). The RMSEA for the hygienic food preparation and storage measure was more moderate (0.098; CI 0.070–0.127), but CFI and TLI were weaker (CFI = 0.886, TLI = 0.848). Model fit for the provision of safe play environment measure was statistically moderate for RMSEA and strong for CFI and TLI (RMSEA = 0.080, CI 0.057–0.103; CFI = 0.978; TLI = 0.972). All three measures demonstrate strong theoretical fit. The finalized tool for all three measures is demonstrated in Appendix A.

### 3.5. Hygiene Behavior Scores

#### 3.5.1. Measure 1 Scores: Hygienic Food Preparation and Storage

Mean scores for the hygienic food preparation and storage measure were 4.30 for the ‘personal beliefs’ factor and 3.93 for the ‘social opportunity’ factor (Table 5). Neither the scores for the ‘personal beliefs’ factor nor the ‘social opportunity’ factor were significantly related to the observable outcomes of the presence of a hygienic food preparation space or hygienic food storage.

#### 3.5.2. Measure 2 Scores: Handwashing at Key Times

The mean scores for the handwashing at key times measure were 4.07 for the ‘physical opportunity’ factor, 3.77 for the ‘social opportunity’ factor, and 4.56 for the ‘beliefs and self-efficacy’ factor (Table 5). Participants able to list key handwashing times scored significantly higher in the ‘beliefs and self-efficacy’ factor; scores for that same latent factor did not differ significantly for any other outcomes of interest. Scores for the ‘social opportunity’ factor were significantly inversely related to the observed presence of soap and water at a handwashing area at time of survey, such that women with lower scores were more likely to exhibit those outcomes. Scores for that same factor were also significantly related to the self-reported use of soap at last time of handwashing for both the mother and the CU2.

#### 3.5.3. Measure 3 Scores: Provision of Safe Play Environment

Mean scores for the provision of safe play environment measure were 3.83 for the ‘perceptions of animal feces’ factor, 3.28 for the ‘social opportunity’ factor, and 4.56 for the ‘reflective motivation’ factor (Table 5). The ‘perceptions of animal feces’ factor was significantly related to the presence of a safe play environment by our overall, operational definition. When we broke the definition down by individual features, scores for the ‘social opportunity’ factor were significantly related to both the presence of human feces and to ‘other harms’, such as sharp objects, in the play environment. Additionally, women who reported the floor as the most recent location of CU2 defecation had significantly higher ‘social opportunity’ scores. Scores for the ‘reflective motivation’ factor were significantly higher for households that did not have other harms present in the play environment. 

## 4. Discussion

We developed a set of three measures, which provide deeper understanding of the latent factors that determine hygienic food preparation and storage, handwashing at key times, and provision of safe play environment behaviors as they relate to environmental pathogen exposure and growth faltering. A theory-informed mixed-methods approach was used to create the three measures, whose structure was developed using EFA and evaluated using CFA. The final hygienic food preparation and storage measure contained a total of 10 items in the ‘personal beliefs’ and ‘social opportunity’ latent factors; the handwashing at key times measure exhibited 15 total items in the ‘physical opportunity’, ‘social opportunity’, and ‘beliefs and self-efficacy’ latent factors; the provision of safe play environment measure demonstrated 14 items across three factors titled ‘perceptions of animal feces’, ‘social opportunity’, and ‘reflective motivation’. These three measures provide an opportunity to gauge the unobservable, latent factors which influence the uptake and application of caregiver hygiene behaviors specific to rural Kenya [21,43]. In applying those measures, we found that ‘social opportunity’ factors held a strong influence over many key outcomes in two of three measures; the knowledge-related ‘beliefs and self-efficacy’ factor in the handwashing at key times measure significantly correlated with other knowledge items but not of actionable behavior; many challenges remain both in defining and quantifying hygienic food preparation and storage; and a participant’s ‘perceptions around animal feces’ factor score was related to the presence of a safe play environment for the CU2.

Each of the three measures included a ‘social opportunity’ factor within the factor structure, and these factors both produced the greatest variability in responses and were statistically significantly related to the most outcomes of interest across all measures (Table 5). The role of social elements in sanitation and hygiene behaviors has long been considered influential [44,45,46,47]. In their eleven-country formative research review, Curtis et al. found status and affiliation to be two of the most commonly cited motivators for handwashing, along with aspects such as disgust, comfort, and fear [45]. 

The score for the ‘social opportunity’ factor of the handwashing at key times measure, unlike the scores relating to ‘reflective motivation’ or ‘beliefs and self-efficacy’, was significantly related to observable practices, such as the presence or absence of soap and water at a handwashing area at time of survey, as well as the self-reported use of soap at last time of handwashing for both the mother and the CU2. The ‘social opportunity’ factor scores were inversely related to presence of soap and water at handwashing stations, such that women with higher scores (i.e., women who were more likely to agree that most people in her community were practicing good handwashing) were less likely to have soap and water present at their own handwashing station at the time of the survey. One potential explanation lies in the “zero contribution thesis”, a theory which states that any self-interested person will not necessarily contribute to the public good; in other words, a person who views themselves to be protected by the actions of others may not be driven to perform that behavior themselves [48,49]. Thus, women who believed that others in their community were practicing good handwashing may have felt less personal motivation to practice good handwashing themselves. Studies that investigate the role of social influences on the provision of safe play environments and hygienic food practices are limited.

Participants with higher ‘beliefs and self-efficacy’ factor scores in the handwashing measure were also significantly more likely to list at least five of six key handwashing times unaided, which is unsurprising as these are both related to knowledge. However, even though the items associated with the latent factor ‘beliefs and self-efficacy’ reflect knowledge that failure of handwashing at key times is a pathway to causing outcomes of illness, participants’ scores on that factor did not differ significantly for any actionable outcomes of interest (for example cleanliness of child’s hands or presence of soap or water at water station). Our research therefore suggests that knowledge relating to the benefits of handwashing or of the potential health consequences stemming from the failure to do so did not necessarily drive or inhibit the performance of handwashing behaviors. These results align with findings from previous studies showing that handwashing knowledge and action are by no means one in the same [50,51]. Globally, knowledge of the benefits of handwashing are high yet practice often remains low [46]. Understanding of latent factors may therefore prove highly valuable in addressing the non-knowledge drivers and barriers to handwashing.

Neither the ‘personal beliefs’ factor nor the ‘social opportunities’ factor were significantly related to the observable outcomes of interest for the hygienic food preparation and storage measure. This is likely due to small populations for both outcomes, with only 14 of 267 households having hygienic food preparation spaces by our operational definition, and only 72 households having previously-prepared food available for storage practices observation at time of surveying. Further exploration of measures like these along with the creation of standard but context-flexible definitions for what defines a hygienic food preparation space, safe food practices, and hygienic food storage is needed for the advancement of this field of research. The topic of food hygiene, both in terms of immediately-consumed and previously-prepared foods, has received relatively little research attention considering that it is a major transmission route for pathogens [46]. Contamination of food has been associated with diarrheal diseases among children fed complementary foods [52]. A study in nearby Kisumu, Kenya found that 71% of all oral contact events for infants aged 3–9 months were related to caregiver feeding [53]. It is therefore not surprising that researchers have found that food hygiene practices may reduce transmission of diarrhea-causing pathogens by 15–70% [54]; food contamination by microbes could be reduced through proper and hygienic food handling and preparation practices including reheating and covering of food [55]. While many physical opportunity constraints in low-resource settings—such as the lack of access to refrigeration and the time- and cost-intensive nature of reheating foods—pose problems outside the scope of small-scale studies, other more manageable behavior changes, such as the order in which raw foods are prepared and storing foods in covered containers away from flies which are addressed in our measure’s items, may still make a substantial impact on health and stunting outcomes in children.

Scores for the ‘perceptions around animal feces’ factor (safe play environment measure) were significantly higher for caregivers who provided operationally-defined safe play environments to CU2. The items in this factor contained both opportunity- and motivation-based items; these findings suggest that participants who believed that most people in their communities had animal feces present in their homes and communities, while also recognizing that chicken, dog, and cow/goat feces can cause illness, were more likely to provide a play space to their CU2 that was free of human and animal feces, garbage, and other harms. Animal feces likely play an underestimated and significant role in the health of young children and all children in low- and middle-income countries (LMIC) [11,56]. Pathogens spread via animal feces can have severe adverse effects for both mother and fetus during pregnancy, and some pathogens have already been shown to be directly related to outcomes of growth stunting [11,56]. Especially as contact between humans and animals is often more frequent in LMIC and animal presence in the domestic environment is more common [57], understanding factors relating to social influences and personal beliefs surrounding the hazards of animal feces will likely prove critical in finding a solution in reducing the burden of these zoonotic pathogens. 

### Strengths and Limitations

The use of the COM-B model as a theoretical foundation, alongside other validated tools and methods throughout all three research phases, is a key strength of this study. A primary strength of the developed measures is their wide applicability. Although this research was focused around growth stunting and its prevention, hygiene behavior change is influential over a wide variety of health outcomes. Good hygiene practices may be influential in reducing the incidence of diarrhea and related morbidities and mortalities, the transmission of foodborne and zoonotic diseases, and EED, all of which are health outcomes closely related to growth stunting [11,12,13].

The qualitative data used to inform the baseline survey and the development of the intervention, while contextually strong and relevant to this sub-study, was not designed explicitly for the development of these measures. There are other aspects of hygiene, such as child feces disposal or water treatment behaviors, that are highly relevant to stunting which were not included; inclusion and exploration of these factors should be considered for future studies.

Statistical fit, using RMSEA as the primary fit statistic, was weak or poor for some of the EFA and CFA models. Small sample sizes (n = 135 for the EFA and for the CFA) may have impacted the results. There is no consensus on sample size for factor analysis [34]. Based on one suggestion to have approximately 10 people per item in the model, our sample size is large enough to be useful, but potentially too small to be generalizable [34,41]. However, our results remain theoretically strong and are backed by the use of validated tools, such as problem and solution trees and a logframe, throughout the process [28,29].

These measures were developed using the data collected from women in Homa Bay and Migori Counties of western Kenya; the results are reflective of those lived experiences, but may not necessarily be applicable among other populations within Kenya or in other countries. Piloting of these measures before use is therefore strongly recommended. 

Finally, the items related to responsive feeding behaviors of CU2 and to porridge thickness (related to caloric density of food) could not be used in our measure development as the number of items were too few and the response variability too low (Appendix B). However, we recognize these and other nutrition- and feeding-related factors to be critical to the successful implementation of stunting interventions. We are expanding measure development to these areas.

## 5. Conclusions

Through a three-phase development and validated process, we developed three measures to quantify the unobservable behavioral antecedents which influence the uptake and application of maternal hygiene behaviors related to environmental pathogen exposures which may contribute to stunting. These measures can also be applied to the study of the many other health outcomes which are affected by hygienic food preparation and storage, handwashing, and play environment, such as diarrhea-related morbidities and mortalities, foodborne and zoonotic diseases, and EED. Similar locally-developed measures have been used previously to gain a deeper understanding of socio-contextual dimensions and implications [21,23,58]; we aim to provide that level of quantitative introspection for maternal hygiene aspects of stunting interventions. The three measures could be used alone or in unison prior to intervention rollout to ensure that it addresses caregivers’ perceived capabilities, motivations, and opportunities throughout intervention design. These measures also provide researchers with the ability to better evaluate the effectiveness of those interventions on a level which is deeper than self-reporting or observed outcomes alone. Notwithstanding promise for future use, these measures are deeply entrenched in a specific context and should be validated prior to use with other study populations or within other geographical regions.

A 2011 review listed measuring hygiene behaviors as one of the top research priorities in the hygiene field [46], yet recent failure of large-scale hygiene studies to reveal impacts on stunting suggest that our understanding of hygiene must go beyond handwashing with soap [59]. An approach using these measures would allow for the development of interventions which address more than just hardware related to hygienic food preparation and storage, handwashing at key times, and provision of safe play environments, but that also address the social and motivational influences on caregivers that may impact the performance of, or failure to perform, a behavior through tailored messages and activities.

## Figures and Tables

**Table 1:** Operational definitions for hygiene-related outcomes of interest.

Outcome of Interest	Operational Definition
Hygienic food preparation space	A hygienic food preparation space will be defined as one which has at least three of the five features:
	○ Presence of a food preparation surface that is cleanable (e.g., one which is not wood or another porous material)
	○ Presence of a food preparation surface that is elevated off the floor
	○ Preparation area is not accessible by animals
	○ Clean utensils
	○ Stored in a space that is not accessible by animals
	○ Stored in a dry space
	○ Visibly free of dirt/debris
	○ Handwashing station can be found within 10 m of the food preparation space
Hygienic storage of previously-prepared foods ^1^	Hygienic storage of foods which have been previously-prepared and are intended for later consumption will be defined as one which has all four features:
	○ Food is not accessible by animals
	○ Food is not accessible by young children
	○ Food is covered
	○ Food is free of flies
Key handwashing times	Awareness of key handwashing times will be defined as the ability to list at least five of the six key handwashing times:
	○ Before food preparation
	○ Before eating
	○ Before feeding child under two years of age
	○ After defecating
	○ After cleaning child feces
	○ After cleaning animal feces
Safe play environment	A safe play environment will be defined as one which has all four features:
	○ Free of human feces
	○ Free of animal feces
	○ Free of garbage/household waste
	○ Free of sharp objects and other potential harms

^1^ Formative work showed that refrigeration is not a consistently available food storage option in this region of rural Kenya; this definition reflects food storage methods which may be attainable to all households in this context (further intervention methodology and results are forthcoming).

**Table 2:** Demographic characteristics of participants by population used for exploratory factor analysis (EFA) and confirmatory factor analysis (CFA).

Characteristics	All Participants		EFA Population		CFA Population	
	n = 270		n = 135		n = 135	
Has child under two years of age *	170	63%	86	64%	84	62%
Currently pregnant	31	11%	10	7%	21	16%
Currently lactating	132	49%	69	51%	63	47%
Currently married	239	89%	118	87%	121	89%
County of residence *						
Homa Bay County	105	39%	53	39%	52	39%
Migori County	165	61%	82	61%	83	61%
Education *						
None	10	4%	7	5%	3	2%
Some primary school (>grade 9)	213	79%	104	77%	109	81%
Some secondary school (grade 9–12)	43	16%	21	16%	22	16%
Beyond secondary school (>grade 12)	4	1%	3	2%	1	1%
Primary drinking water source location						
Surface water	138	51%	70	52%	68	50%
Borehole/tubewell	64	24%	30	22%	34	25%
Public tap/standpipe	25	9%	11	8%	14	10%
Rainwater collection	20	7%	11	8%	9	7%
Other	23	9%	13	10%	10	7%
Household latrine access						
Yes	120	44%	53	39%	67	50%
No	150	56%	82	61%	68	50%
Food insecurity (HFIAS) ^1^						
Experienced anxiety and uncertainty about food supply in last 30 days ^1,2^	229	85%	113	84%	116	86%
Reported insufficient quality of food supply in last 30 days ^1,2^	249	92%	123	91%	126	93%
Reported insufficient food intake and its physical consequences in last 30 days ^1,2^	247	91%	122	90%	125	93%

* Indicates a characteristic that was used to create a stratified random sample. ^1^ Coates, J., Swindale, A., & Bilinsky, P. [22]. ^2^ These results represent households experiencing any of the conditions at any level of severity in each of the Household Food Insecurity Access-related domains (e.g., these results reflect responses to occurrence questions only and do not reflect the proscribed HFIAS frequency component) [22]. For this reason, these results may differ from other reports in this region.

**Table 3:** Key hygiene behaviors of interest by population used for exploratory factor analysis (EFA) and confirmatory factor analysis (CFA).

Characteristics	n (Total Population)	Percentage
Hygienic food preparation and storage		
Presence of hygienic food preparation space ^1^	14 (267)	5%
Previously prepared food is stored hygienically ^1^	30 (72)	42%
Handwashing at key times		
Participant able to list five or six key handwashing times ^1^	70 (270)	26%
Water present at handwashing station	30 (258)	12%
Soap present at handwashing station	19 (258)	7%
Child’s hands are clean (clean palms and finger pads observed on both hands) ^2^	62 (150)	41%
Soap used last time the child’s hands were washed (self-reported)	144 (170)	85%
Soap used last time the mother’s hands were washed (self-reported)	236 (270)	87%
Provision of safe play environment		
Presence of safe play environment ^1^	55 (161)	34%
Presence of garbage in play environment	70 (161)	43%
Presence of human feces in play environment	20 (161)	12%
Presence of animal feces in play environment	86 (161)	53%
Presence of other harms (i.e., sharp objects) in play environment	63 (161)	39%
Child defecated on floor at last time of defecation (self-reported)	88 (170)	52%

^1^ Operational definition is outlined in Table 1. ^2^ Handwashing behavior measure adapted from an observed handwashing study [37]. Total population varies due to some households not consenting to certain observations, or because some households did not currently have a child under two to observe hand cleanliness or play environment safety. Only households with previously-prepared food at time of survey could be considered for hygienic food storage qualities.

**Table 4:** Exploratory and confirmatory factor analysis results for hygienic food preparation and storage behaviors measure, handwashing behaviors measure, and safe play environment provision measure (n_EFA_ = 135; n_CFA_ = 135).

Factors and Associated Items	Item Number	COM-B Component ^1^	Final EFA Factor Loading	Final CFA Factor Loading
**Measure 1: Hygienic Food Preparation and Storage**				
Factor 1: Social Opportunity				
Most people in my community prepare food safely.	E.1.2	O	0.642	0.519
Most people in my community cover prepared food in between meals.	E.1.12	O	0.904	0.869
Most people in my community reheat previously cooked food before feeding it to their families.	E.1.13	O	0.782	0.712
Factor 2: Personal Beliefs				
It is not necessary to reheat food for meals prepared early in the day.	E.1.4	C	0.698	0.787
It is okay to cut vegetables with the same knife just after I cut raw chicken or fish.	E.1.5	C	0.618	0.732
It is beneficial to wash food before preparation.	E.1.6	M	0.622	0.567
It is beneficial to store food in a covered container.	E.1.7	M	0.642	0.388
It is safe to consume meat when the juices run red or pink.	E.1.10	M	0.814	0.846
Food that has not been covered is still safe to consume.	E.1.14	M	0.697	0.792
I would feel confident to demonstrate preparation of food for children under two to others in my community.	E.1.56	C	0.595	0.513
**Measure 2: Handwashing at Key Times**				
Factor 1: Physical Opportunity				
I always have water for handwashing.	E.1.31	O	0.824	0.656
It is possible for me to buy soap for handwashing.	E.1.32	O	0.363	0.689
Sometimes I don’t wash my hands because I don’t have enough time.	E.1.34	O	0.633	0.528
Factor 2: Social Opportunity				
Most people in my community have soap.	E.1.35	O	0.662	0.624
Most people in my community use soap every time they wash their hands.	E.1.36	O	0.854	0.692
Most people in my community wash their hands after defecating.	E.1.37	O	0.917	0.851
Most people in my community wash their hands before preparing food.	E.1.38	O	0.884	0.903
Most people in my community wash their hands before feeding a young child.	E.1.39	O	0.936	0.910
Most people in my community wash their hands before eating.	E.1.40	O	0.730	0.825
Most people in my community wash the hands of a child under two years old before the child eats.	E.1.41	O	0.724	0.858
Factor 3: Beliefs and Self-Efficacy				
It is important for me to have soap available for handwashing.	E.1.33	M	0.512	0.557
Not washing my hands before preparing food can make my child sick.	E.1.42	M	0.796	0.929
Not washing my hands after touching the feces of my young child can cause me to become ill.	E.1.43	M	0.942	0.813
Washing your hands after you change your baby’s nappies or diapers can prevent you and your child from becoming ill.	E.1.44	M	0.359	0.385
I would feel confident to demonstrate excellent hand washing techniques to others in my community.	E.1.57	C	0.540	0.572
**Measure 3: Provision of Safe Play Environment**				
Factor 1: Perceptions around Animal Feces				
Most people in this community have animal feces (including chicken feces) present in their compound.	E.1.17	O	0.506	0.710
Most people in this community have animal feces (including chicken feces) present in their house.	E.1.18	O	0.424	0.570
I find it disgusting when animal feces (including chicken feces) are present within a compound.	E.1.27	O	0.583	0.753
Chicken feces can make you sick.	E.1.28	M	0.587	0.646
Dog feces can make you sick.	E.1.29	M	0.682	0.856
Cow/goat feces can make you sick.	E.1.30	M	0.679	0.599
Factor 2: Social Opportunity				
Most people in my community have a designated play area for their young children.	E.1.19	O	0.672	0.356
Most children in this community play in areas that are free from human feces.	E.1.20	O	0.815	0.777
Most children in this community play in areas that are free from animal feces (including chicken feces).	E.1.21	O	0.996	0.939
Most children in this community play in areas that are free from garbage or other wastes.	E.1.22	O	0.929	0.941
Factor 3: Reflective Motivation				
It is possible for me to provide a play space to my child that is free of animal feces (including chicken feces).	E.1.23	M	−0.864	0.911
It is possible for me to provide a play space to my child that is free of human feces.	E.1.24	M	−1.001	0.950
It is possible for me to provide a play space to my child that is free of garbage and other household wastes.	E.1.25	M	−0.924	0.983

^1^ COM-B Model: capability (having the skills necessary to perform the behavior), opportunity (having the social and environmental abilities to perform the behavior), and motivation (having personal and external reasons to perform the behavior) [26]. Hygiene behavior measures are shown in bold font.

**Table 5:** Hygiene behavior measure scores by key outcomes. Numbers are mean score (SD).

**Hygienic Food Preparation and Storage**		**Personal Beliefs**	**Social Opportunity**	
All	n = 270	4.30 (0.74)	3.93 (0.99)	
Presence of hygienic food preparation space ^1^	n = 267			
No		4.31 (0.74)	3.92 (0.99)	
Yes		4.05 (0.84)	4.06 (0.94)	
Previously prepared food is stored hygienically ^1^	n = 72			
No		3.97 (0.84)	4.10 (0.97)	
Yes		4.25 (0.80)	4.14 (1.12)	
**Handwashing at Key Times**		**Physical Opportunity**	**Social Opportunity**	**Beliefs and Self-efficacy**
All	n = 270	4.07 (0.88)	3.77 (1.05)	4.56 (0.58)
Participant able to list key handwashing times ^1^	n = 270			
No		4.08 (0.89)	3.81 (1.05)	4.52 (0.61) *
Yes		4.06 (0.88)	3.66 (1.06)	4.69 (0.47) *
Water present at handwashing station	n = 258			
No		4.06 (0.89)	3.85 (0.98) *	4.53 (0.61)
Yes		4.29 (0.12)	3.40 (1.35) *	4.73 (0.44)
Soap present at handwashing station	n = 258			
No		4.07 (0.88)	3.84 (0.99) *	4.54 (0.60)
Yes		4.37 (0.64)	3.33 (1.47) *	4.75 (0.42)
Child’s hands are visibly clean	n = 150			
No		4.09 (0.83)	3.77 (1.05)	4.44 (0.62)
Yes		4.24 (0.88)	3.77 (1.10)	4.58 (0.61)
Soap used last time child’s hands washed	n = 170			
No		3.87 (0.89)	3.21 (1.22) **	4.39 (0.63)
Yes		4.10 (0.89)	3.85 (1.03) **	4.55 (0.60)
Soap used last time mother’s hands washed	n = 270			
No		3.56 (0.94) **	3.31 (0.91) **	4.51 (0.50)
Yes		4.15 (0.85) **	3.84 (1.05) **	4.57 (0.59)
**Provision of Safe Play Environment**		**Perceptions of Animal Feces**	**Social Opportunity**	**Reflective Motivation**
All	n = 270	3.83 (0.57)	3.28 (1.32)	4.56 (0.92)
Presence of safe play environment ^1^	n = 161			
No		3.70 (0.64) *	3.23 (1.38)	4.58 (0.85)
Yes		3.95 (0.50) *	3.30 (1.34)	4.74 (0.71)
Presence of garbage in play environment	n = 161			
No		3.70 (0.58)	3.35 (1.39)	4.70 (0.70)
Yes		3.85 (0.62)	3.18 (1.35)	4.59 (0.88)
Presence of human feces in play environment	n = 161			
No		3.62 (0.64)	2.60 (1.44) *	4.68 (0.86)
Yes		3.81 (0.60)	3.35 (1.33) *	4.63 (0.80)
Presence of animal feces in play environment	n = 161			
No		3.71 (0.62)	3.26 (1.39)	4.63 (0.85)
Yes		3.87 (0.58)	3.24 (1.35)	4.64 (0.77)
Presence of other harms in play environment	n = 161			
No		3.69 (0.57)	2.98 (1.40) *	4.48 (0.91) *
Yes		3.84 (0.62)	3.43 (1.32) *	4.74 (0.72) *
At last defecation, child defecated on floor	n = 170			
No		3.83 (0.43)	3.00 (1.30) *	4.68 (0.73)
Yes		3.78 (0.72)	3.44 (1.35) *	4.54 (0.97)

Mean score (SD); * *p*-value < 0.05; ** *p*-value < 0.005; ^1^ Operational definition outlined in Table 1. Total population varies due to some households not consenting to certain observations, or because some households did not currently have a child under two to observe hand cleanliness or play environment safety. Only households with previously-prepared food at time of survey could be considered for hygienic food storage qualities. Hygiene behavior measures and their corresponding factors are shown in bold font.

## Supplementary Materials

The following are available online at http://www.mdpi.com/1660-4601/15/9/1994/s1, Table S1: All original items related to hygienic food preparation and storage and frequency of responses. Supplementary Table S2: All original items related to the provision of a safe play environment and frequency of responses. Supplementary Table S3: All original items related to handwashing at key times and frequency of responses. Supplementary Table S4: All original items related to responsive feeding practices and preparation of porridge of sufficient caloric density, and frequency of responses.

## Appendix A

The tool for the three hygiene behavior measures is provided below; items are organized by factor within each measure.

**Table A1:** Hygiene behaviors tool, organized by measure and factor.

Caregiver Hygiene Behavior Measures	1 Strongly Disagree	2 Somewhat Disagree	3 Neither Agree nor Disagree	4 Somewhat Agree	5 Strongly Agree
Hygienic Food Preparation and Storage					
**Social Opportunity**					
Most people in my community prepare food safely.					
Most people in my community cover prepared food between meals.					
Most people in my community reheat previously cooked food before feeding it to their families.					
**Personal Beliefs**					
It is not necessary to reheat food for meals prepared early in the day.					
It is okay to cut vegetables with the same knife just after I cut raw chicken or fish.					
It is beneficial to wash food before preparation.					
It is beneficial to store food in a covered container.					
It is safe to consume meat when the juices run red or pink.					
Food that has not been covered is still safe to consume.					
I would feel confident to demonstrate preparation of food for children under two to others in my community.					
**Handwashing**					
**Physical Opportunity**					
I always have water for handwashing.					
It is possible for me to buy soap for handwashing.					
Sometimes I don’t wash my hands because I don’t have enough time.					
**Social Opportunity**					
Most people in my community have soap.					
Most people in my community use soap every time they wash their hands.					
Most people in my community wash their hands after defecating.					
Most people in my community wash their hands before preparing food.					
Most people in my community wash their hands before feeding a young child.					
Most people in my community wash their hands before eating.					
Most people in my community wash the hands of a child under two years old before the child eats.					
**Beliefs and Self-efficacy**					
It is important for me to have soap available for handwashing.					
Not washing my hands before preparing food can make my child sick.					
Not washing my hands after touching the feces of my young child can cause me to become ill.					
Washing your hands after you change your baby’s nappies or diapers can prevent you and your child from becoming ill.					
I would feel confident to demonstrate excellent hand washing techniques to others in my community.					
**Safe Play Environment**					
**Perceptions around Animal Feces**					
Most people in this community have animal feces (including chicken feces) present in their compound.					
Most people in this community have animal feces (including chicken feces) present in their house.					
I find it disgusting when animal feces (including chicken feces) are present within a compound.					
Chicken feces can make you sick.					
Dog feces can make you sick.					
Cow/goat feces can make you sick.					
**Social Opportunity**					
Most people in my community have a designated play area for their young children.					
Most children in this community play in areas that are free from human feces.					
Most children in this community play in areas that are free from animal feces (including chicken feces).					
Most children in this community play in areas that are free from garbage or other wastes.					
**Reflective Motivation**					
It is possible for me to provide a play space to my child that is free of animal feces (including chicken feces).					
It is possible for me to provide a play space to my child that is free of human feces.					
It is possible for me to provide a play space to my child that is free of garbage and other household wastes.					

## Appendix B

Responsive feeding and porridge thickness items for which factor structures could not be determined due to a small number of items and low variability in responses. Both behaviors were dropped from further exploration.

**Table A2:** All items related to caregiver behaviors surrounding responsive feeding practices and preparation of porridge of sufficient caloric density.

Items	COM-B Component
Responsive Feeding Practices	
Infants show signs of hunger when they start crying.	C
Infants show signs of hunger when they start reaching for their mother’s breast.	C
Infants show signs of hunger when they put an object in their mouth.	C
I try to feed my child when he/she looks at other people who are eating.	C
I try to feed my child when he/she moves mouth and tongue as if eating.	C
I try to feed my child when he/she drools or spits.	C
I try to feed my child when he/she puts other objects into his/her mouth.	C
Preparation of Porridge of Sufficient Caloric Density	
It does not matter how thick or thin my child’s porridge is.	C
Thick porridge has more nutrients than thin porridge.	C
Thick porridge is a choking hazard.	C
Thick porridge will give my child stomach problems.	M
