# Supplementary material for: Development and Application of Novel Caregiver Hygiene Behavior Measures Relating to Food Preparation, Handwashing, and Play Environments in Rural Kenya
Source: Int J Environ Res Public Health. 2018 Sep 13;15(9):1994. doi: 10.3390/ijerph15091994 (PMC6163645; doi:10.3390/ijerph15091994)
Supplement: Supplementary file 1 [file ijerph-15-01994-s001.pdf]

International Journal of Environmental Research and Public Health

Supplementary Materials for:

Development and Application of Novel Caregiver Hygiene Behavior Measures Relating to Food Preparation, Handwashing, and Play Environments in Rural Kenya

Breanna K. Wodnik[1,*], Matthew C. Freeman[2], Anna S. Ellis[2], Emily Awino Ogutu[2], Amy Webb-Girard[1], Bethany A. Caruso[2]

[1] Hubert Department of Global Health, Rollins School of Public Health, Emory University, Atlanta, GA 30322, USA; bwodnik@emory.edu; awebb3@emory.edu

[2] Department of Environmental Health, Rollins School of Public Health, Emory University, Atlanta, GA 30322, USA; matthew.freeman@emory.edu; anna.s.ellis@emory.edu; emily.awino@yahoo.com; bethany.caruso@emory.edu

[*] Correspondence: bwodnik@emory.edu

Supplementary Table S1. All original items related to hygienic food preparation and storage and frequency of responses (organized by COM-B component).

Hygienic Food Preparation and Storage n=270									
	**Likert Scale** (1 = strongly disagree, 5 = strongly agree)					**Descriptive Statistics**			**COM-B Component**
**Item**	**1**	**2**	**3**	**4**	**5**	**Mean**	**Kurtosis**	**Skewness**	
E.1.4 It is not necessary to reheat food for meals prepared early in the day.	151	15	7	28	69	2.44	1.45	0.55	Capability Psychological
E.1.5 It is okay to cut vegetables with the same knife just after I cut raw chicken or fish.	224	9	2	13	22	1.52	5.93	2.16	Capability Psychological
E.1.8 I re-heat previously cooked food every time before feeding it to my family.	3	3	1	15	248	4.86	30.45	-5.08	Capability Psychological
E.1.56 I would feel confident to demonstrate preparation of food for children under 2 to others in my community.	7	4	5	31	223	4.70	13.94	-3.29	Capability Physical
E.1.2 Most people in my community prepare food safely.	26	13	50	42	139	3.94	2.83	-1.02	Opportunity Social
E.1.12 Most people in my community cover prepared food in between meals.	16	18	66	40	130	3.93	2.63	-0.83	Opportunity Social
E.1.13 Most people in my community reheat previously cooked food before feeding it to their families.	15	12	77	42	124	3.92	2.73	-0.79	Opportunity Social
E.1.1 Preparing food in a clean place is important.	0	0	1	2	267	4.99	133.23	-11.05	Motivation Reflective
E.1.3 Reheating previously cooked food makes it less likely to make you sick.	38	10	3	26	193	4.21	3.65	-1.55	Motivation Reflective
E.1.6 It is beneficial to wash food before preparation.	5	4	4	17	240	4.79	19.00	-3.97	Motivation Reflective
E.1.7 It is beneficial to store food in a covered container.	7	3	2	19	239	4.78	18.90	-4.00	Motivation Reflective
E.1.9 It is important when cooking soup or other liquid foods to bring them to a full boil.*	174	3	4	13	55	2.08	2.09	1.00	Motivation Reflective
E.1.10 It is safe to consume meat when the juices run red or pink.*	32	4	3	15	195	4.35	4.73	-1.88	Motivation Reflective

E.1.11 Thorough cooking of food makes it safe to eat.	1	0	1	5	263	4.96	118.56	-9.99	Motivation Reflective
E.1.14 Food that has NOT been covered is still safe to consume.	154	29	14	26	47	2.20	2.02	0.85	Motivation Reflective
E.1.15 Food that has not been covered between mealtimes can make my family sick.	24	3	8	33	202	4.43	6.24	-2.15	Motivation Reflective
E.1.16 If food has been sitting out for more than 4 hours, it can make my family sick if they eat it.	84	21	19	40	106	3.23	1.32	-0.27	Motivation Reflective
E.1.58 The reason I COOK foods thoroughly is because they taste better warm.	1	0	1	16	252	4.92	60.16	-6.53	Motivation Reflective
E.1.59 The reason I COOK foods thoroughly is to prevent sickness in my family.	0	1	2	10	257	4.94	44.29	-5.98	Motivation Reflective
E.1.60 The reason I REHEAT foods is because they taste better warm.	2	2	1	10	255	4.90	44.73	-6.20	Motivation Reflective
E.1.61 The reason I REHEAT foods is to prevent sickness in my family.	0	0	3	9	258	4.93	51.04	-6.64	Motivation Reflective

*This question was added later; 249 participants responded

Supplementary Table S2. All original items related to the provision of a safe play environment and frequency of responses (organized by COM-B component).

**Provision of Safe Play Environment**<br>n=270									
	**Likert Scale**<br>(1 = strongly disagree, 5 = strongly agree)					**Descriptive Stats**			**COM-B Component**
**Item**	**1**	**2**	**3**	**4**	**5**	**Mean**	**Kurtosis**	**Skewness**	
E.1.17 Most people in this community have animal feces (including chicken feces) present in their COMPOUND.	23	6	30	35	176	4.24	4.29	-1.58	Opportunity Social
E.1.18 Most people in this community have animal feces (including chicken feces) present in their HOUSE.	59	13	38	43	117	3.54	1.79	-0.60	Opportunity Social
E.1.19 Most people in my community have a designated play area for their young children.	104	15	44	33	74	2.84	1.36	0.10	Opportunity Social
E.1.20 Most children in this community play in areas that are free from human feces.	52	15	31	34	138	3.71	1.97	-0.77	Opportunity Social
E.1.21 Most children in this community play in areas that are free from animal feces (Including CHICKEN feces).	73	32	27	34	104	3.24	1.37	-0.23	Opportunity Social
E.1.22 Most children in this community play in areas that are free from garbage or other wastes.	69	29	24	35	113	3.35	1.42	-0.35	Opportunity Social
E.1.26 I find it disgusting when animal feces (including CHICKEN feces) are present inside a house.	8	5	3	15	239	4.75	15.32	-3.61	Opportunity Social
E.1.27 I find it disgusting when animal feces (including CHICKEN feces) are present within a compound.	10	4	2	10	244	4.76	15.47	-3.69	Opportunity Social
E.1.23 It is possible for me to provide a play space to my child that is free of ANIMAL feces (including CHICKEN feces).	13	16	9	29	203	4.46	5.93	-2.05	Motivation Reflective
E.1.24 It is possible for me to provide a play space to my child that is free of HUMAN feces.	11	7	8	21	223	4.62	9.61	-2.75	Motivation Reflective

E.1.25 It is possible for me to provide a play space to my child that is free of garbage and other household wastes.	8	13	6	23	220	4.61	8.63	-2.58	Motivation Reflective
E.1.28 Chicken feces can make you sick.	18	4	10	12	226	4.57	8.04	-2.54	Motivation Reflective
E.1.29 Dog feces can make you sick.	2	0	2	7	259	4.93	66.02	-7.52	Motivation Reflective
E.1.30 Cow / goat feces can make you sick.	20	6	10	13	221	4.51	6.86	-2.31	Motivation Reflective

Supplementary Table S3. All original items related to handwashing at key times and frequency of responses (organized by COM-B component).

**Handwashing at Key times** n=270									
	**Likert Scale** (1 = strongly disagree, 5 = strongly agree)					**Descriptive Statistics**			**COM-B Component**
**Item**	**1**	**2**	**3**	**4**	**5**	**Mean**	**Kurtosis**	**Skewness**	
E.1.57 I would feel confident to demonstrate excellent hand washing techniques to others in my community.	9	4	2	29	226	4.70	14.00	-3.37	Capability Physical
E.1.31 I always have water for handwashing.	6	8	0	20	236	4.75	15.24	-3.58	Opportunity Physical
E.1.32 It is possible for me to buy soap for handwashing	21	10	1	34	204	4.44	6.22	-2.16	Opportunity Physical
E.1.34 Sometimes I don't wash my hands because I don't have enough time.	122	8	1	33	106	2.97	1.10	-0.02	Opportunity Physical
E.1.35 Most people in my community have soap.	49	16	41	53	111	3.60	1.99	-0.67	Opportunity Social
E.1.36 Most people in my community use soap EVERY TIME they wash their hands.	55	20	53	57	85	3.36	1.79	-0.43	Opportunity Social
E.1.37 Most people in my community wash their hands after defecating.	50	11	52	51	106	3.56	2.01	-0.64	Opportunity Social
E.1.38 Most people in my community wash their hands before preparing food.	34	14	55	55	112	3.73	2.42	-0.79	Opportunity Social
E.1.39 Most people in my community wash their hands before feeding a young child.	28	14	58	47	123	3.83	2.57	-0.86	Opportunity Social

E.1.40 Most people in my community wash their hands before eating.	10	7	50	31	172	4.29	4.14	-1.41	Opportunity Social
E.1.41 Most people in my community wash the hands of a CHILD under 2 years old before the child eats.	15	15	56	37	147	4.06	3.03	-1.05	Opportunity Social
E.1.33 It is important for me to have soap available for handwashing.	1	1	1	27	240	4.87	32.81	-4.70	Motivation Reflective
E.1.42 Not washing my hands before preparing food can make my child sick.	10	21	13	23	203	4.44	5.22	-1.89	Motivation Reflective
E.1.43 Not washing my hands after touching the feces of my young child can cause me to become ill.	14	21	12	19	204	4.40	4.87	-1.82	Motivation Reflective
E.1.44 Washing your hands after you change your baby's nappies or diapers can prevent you and your child from becoming ill.	20	8	11	32	199	4.41	5.86	-2.04	Motivation Reflective

Supplementary Table S4. All original items related to responsive feeding practices and preparation of porridge of sufficient caloric density, and frequency of responses (organized by COM-B component).

Responsive Feeding Practices n=270									
	Likert Scale (1 = strongly disagree, 5 = strongly agree)					Descriptive Statistics			COM-B Component
**Item**	**1**	**2**	**3**	**4**	**5**	**Mean**	**Kurtosis**	**Skewness**	
E.1.45 It does not matter how thick or thin my child's porridge is.	90	45	33	60	42	2.70	1.54	0.20	Capability Psychological
E.1.46 Thick porridge has more nutrients than thin porridge.	27	26	12	64	141	3.99	2.93	-1.16	Capability Psychological
E.1.47 Thick porridge will give my child stomach problems	99	38	14	46	73	2.84	1.31	0.14	Capability Psychological
E.1.49 Infants show signs of hunger when they start crying.	40	13	5	30	182	4.11	3.16	-1.37	Capability Psychological
E.1.50 Infants show signs of hunger when they start reaching for their mothers' breast.	10	4	0	15	241	4.75	15.84	-3.72	Capability Psychological
E.1.51 Infants show signs of hunger when they put an object in their mouth.	59	5	3	17	186	3.99	2.45	-1.16	Capability Psychological
E.1.52 I try to feed my child when he or she looks at other people who are eating.*	63	7	2	8	90	3.32	1.18	-0.33	Capability Psychological

E.1.53 I try to feed my child when he or she moves mouth and tongue as if eating.*	58	7	6	11	88	3.38	1.27	-0.39	Capability Psychological
E.1.54 I try to feed my child when he or she drools or spits.*	85	16	2	16	51	2.60	1.31	0.40	Capability Psychological
E.1.55 I try to feed my child when he or she puts other objects into her/his mouth.*	83	8	3	16	60	2.78	1.15	0.20	Capability Psychological
E.1.48 Thick porridge will give my child stomach problems.	59	36	26	43	106	3.37	1.51	-0.37	Motivation Reflective

*This question only asked of mothers with children under the age of 2; 170 participants total.
